# Skin rash caused by EGFR-TKI could be treated successfully by Pien Tze Huang Unguentum Compositum: a case report

**DOI:** 10.7555/JBR.36.20220065

**Published:** 2022-06-28

**Authors:** Mingzi Zhang, Qi Li, Yehong Sun

**Affiliations:** 1 Department of Pharmacy, Shenzhen Traditional Chinese Medicine Hospital, Shenzhen, Guangdong 518033, China; 2 Department of Internal Medicine-Oncology, Shenzhen Traditional Chinese Medicine Hospital, Shenzhen 518033, China

**Keywords:** epidermal growth factor receptor, tyrosine kinase inhibitor, skin rash, Pien Tze Huang, erlotinib

## Abstract

Epidermal growth factor receptor tyrosine kinase inhibitor (EGFR-TKI) plays an important role in cancer therapy. However, EGFR is highly expressed in the skin and gives rise to one of the most concerning issues for the EGFR-TKI treatment, namely skin toxicity. Antibiotics and corticosteroids are usually used to treat the EGFR inhibitor-associated skin rash, with prominent side effects over long-time use. Pien Tze Huang (PZH) Unguentum Compositum is a traditional product for external application which is made of traditional Chinese medicine and oil base. Herein, we reported the case of a 50-year-old man who presented with skin rash on the face, head, and back induced by an EGFR-TKI named erlotinib. By using PZH Unguentum Compositum, we observed that the skin rash was mitigated and eventually disappeared. This case report suggests that PZH Unguentum Compositum may be an effective therapy in treating skin rash caused by EGFR-TKI with fewer side effects.

## Introduction

Epidermal growth factor receptor (EGFR) is a key molecule in investigation of lung cancer, and it is also a target for a new therapeutic strategy for treatment of other tumors ^[ 1– 2] ^. An increasing number of molecular targeting agents such as EGFR-TKIs have been used for the treatment of cancer in recent decades ^[ 3– 4] ^. The toxicity of EGFR-TKIs is entirely different from that of the traditional cytotoxic chemotherapeutic agents ^[ 5] ^. Skin toxicity is the most concerning issue for the EGFR-TKI treatment as EGFR is highly expressed in the skin. Skin toxicity includes rash acneiform, skin fissure and xerosis which are sometimes painful ^[ 6] ^. These side effects also severely decrease the quality of life and potentially affect treatment compliance. Skin rash is common among patients treated with EGFR inhibitors, and the severity of skin rash may correlate with the efficacy of the treatment. Generally, the acne-like skin rash and pruritus are experienced in 1–2 weeks after starting EGFR-TKI treatment. The incidence of skin rash was 73%–99% in erlotinib and the frequency of grade 3 or higher skin rash toxicity was 2%–19% in erlotinib ^[ 6] ^. Although the systemic or topical use of antibiotics and corticosteroid has been proved to be effective in the treatment of the EGFR inhibitor-associated skin rash, they are not suitable for long-term use due to their prominent side effects, including intestinal dysbacteriosis and the decline of immunity ^[ 6] ^.

Traditional Chinese medicine (TCM) is believed to have few side effects and has been used in China for more than 1000 years as remedy. Pien Tze Huang (PZH) is a TCM agent firstly formulated by a court doctor during the Ming Dynasty (circa 1555 AD) in China, containing musk (Shexiang), Calculus Bovis (Niuhuang, ox's gallstone), Shedan (snake's gall) and Panax notoginseng (Tianqi or Sanqi). It is used to treat liver diseases, cancer, and inflammation ^[ 7] ^. The components of PZH possess anti-edema, anti-inflammatory, and anti-thrombotic effects. PZH Unguentum Compositum is a traditional product for external application which is made of Chinese materia medica and oil base. The main ingredients include PZH power, snake tablets, and some oil base. PZH Unguentum Compositum was developed and produced by Zhangzhou Pientzehuang Pharmaceutical Co., Ltd. (Zhangzhou, China). Recent studies have demonstrated that the cream presents potent therapeutic effects in clinical usage of herpes zoster (shingles), herpes simplex, impetigo, folliculitis, and acne. National Medical Products Administration (NMPA) has approved the use of PZH Unguentum Compositum for the above-mentioned indications ^[ 8] ^. Herein, we present a successfully treated case of skin rash caused by erlotinib using PZH Unguentum Compositum, in order to provide a new option for EGFR TKI-induced skin rash.

## Case report

**Figure 1:**
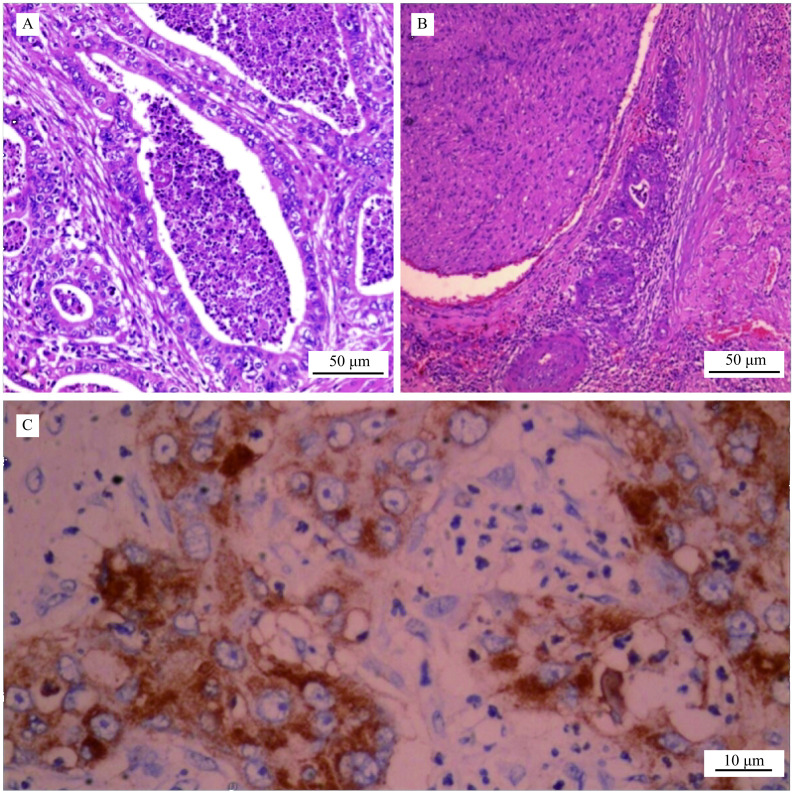
Microscopic view of the tumor (received from pathologist).

In October, 2016, a 50-year-old man who presented with pains in the left upper abdomen during the last 2 weeks was referred to Shenzhen Traditional Chinese Medicine Hospital (Shenzhen, China). Abdominal computed tomography (CT) scan showed the presence of a pancreatic mass, and the probability of cancer is very high, with the cancer antigen 19-9 (CA 19-9) of 310.1 U/mL. Then the patient accepted surgery in the same month and exploratory laparotomy, excision of pancreatic body and tail, splenectomy, and incision of pancreatic duct allowing stones to be removed, and Roux-en-Y anastomosis of pancreas and jejunum were done. The infringed left artery of the stomach can be seen during the operation. Postoperative pathological and immunohistochemical results revealed ductal adenocarcinoma with medium-low differentiation ( ***Fig. 1***). The patient's immunohistochemical report suggested that the tumor was cytokeratin (CK) 7 ^+^, CK8 ^+^, CK19 ^+^, carcino-embryonic antigen ^+^, vimentin ^−^, α-fetoprotein ^−^, synaptophysin ^+^, chromogranin A ^+^, and progesterone receptor (10% positive). The patient was then treated with postoperative intravenous gemcitabine 1.4 g (1000 mg/m ^2^, on days 1 and 8) combined with S-1 60 mg (twice a day, on days 1 to 14) for the first cycle from December 8, 2016. He was treated with the second cycle of chemotherapy on January 6, 2017, but the patient refused the chemotherapy after 3 days of treatment. So, the second cycle of chemotherapy was unfinished.

**Figure 2:**
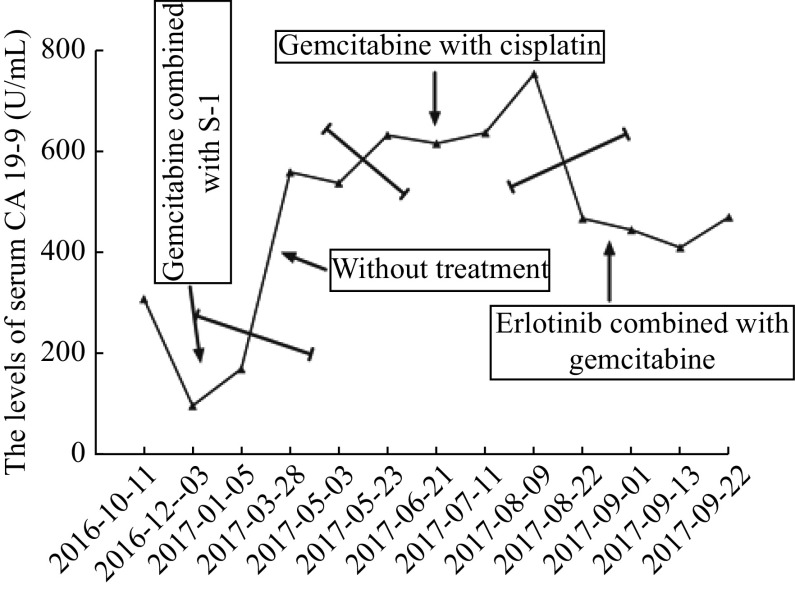
The levels of serum CA 19-9 before and after erlotinib treatment.

In April, 2017, the patient was admitted to our hospital again due to pains in the upper left abdomen for about half a month. The upper abdomen CT scan showed that his soft tissue lesions have appeared in the pancreatic head - jejunal anastomosis area. And CA 19-9 was 560.1 U/mL. Tumor recurrence was diagnosed. The patient was administered cisplatin for 40 mg on day 1, and 30 mg for days 2 to 3, plus gemcitabine 1400 mg (1000 mg/m ^2^, on days 1 and 8) of a 21-day cycle for five cycles from April 14, 2017 to July 20, 2017. After four cycles of chemotherapy, it was evaluated by CT scan as stable disease (SD), but the values of CA 19-9 rose slightly. We decided to give the patient a cycle of chemotherapy as the CT scan indicated SD. Unfortunately, after a cycle of the chemotherapy, CT scan showed a progressive disease. The CA 19-9 level was significantly increased to 754.9 U/mL ( ***Fig. 2***).

**Figure 3:**
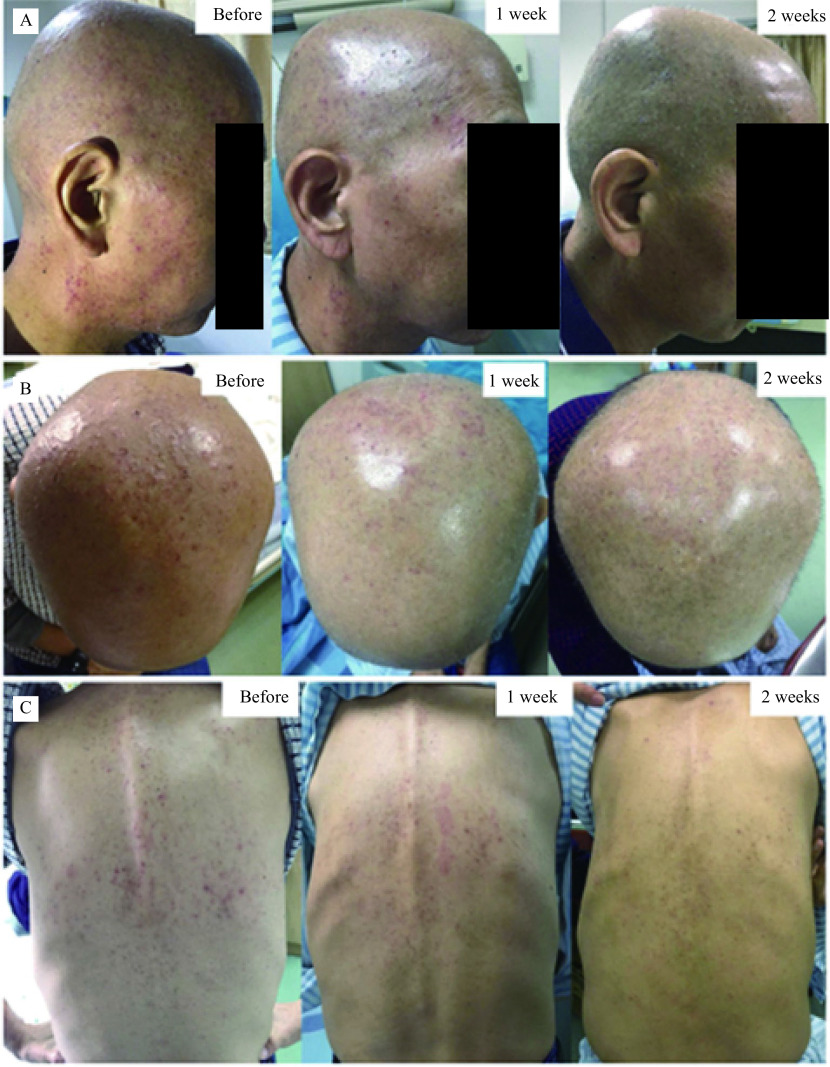
Skin rash on the face (A), head (B), and trunk (C) before and after external use of PZH Unguentum Compositum Cream.

Hence, we started a new regimen of EGFR-TKI, erlotinib (100 mg, qd) combined with chemotherapy of gemcitabine 1.4 g (1000 mg/m ^2^, on days 1 and 8) from August 14, 2017. The patient signed the informed consent for administering erlotinib and gemcitabine, and provided his written informed consent for the accompanying images to be published in this case report. Within the first week of treatment, patients experienced sensory disturbance with erythema and edema. About a week after the initiation of the new regimen, the patient noticed dry skin and skin rash on his face, head and trunk ( ***Fig. 3***). Grade 2 skin rash was diagnosed (NCI-CTC 4.0). Then PZH Unguentum Compositum was prescribed to the patient (three times per day，with suitable amount on the rash each time), and we observed that the skin rash mitigated and almost disappeared after continuous external use for 2 weeks ( ***Fig. 3***). We suggested the patient to keep using the cream since skin rash may reappear at any time.

**Figure 4:**
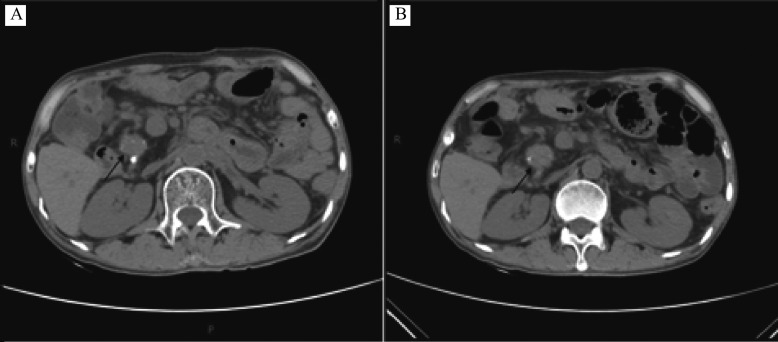
Computed tomography scan images.

After three cycles of erlotinib combined with gemcitabine treatment from August 14, 2017 to September 27, 2017, the CT evaluation indicated SD ( ***Fig. 4***), and the CA 19-9 level was also decreased significantly compared with that before the new regimen was given ( ***Fig. 2***). After that, the patient continued to use erlotinib (100 mg, qd) as maintenance therapy in case of the tumor progressed. However, the patient's son informed us that the disease was out of control by erlotinib in February, 2018 at a follow-up visit, and they had decided to give up therapy for economic reasons. From then on, we lost the contact with the patient.

In total, the patient had undergone erlotinib treatment for 6 months without other major toxic effects except mild and occasional diarrhea (about 2 to 3 times per day).

## Discussion

Erlotinib, an oral EGFR-TKI, is currently widely used as an effective targeted therapy for patients with non-small cell lung cancer (NSCLC) and pancreatic cancer ^[ 1– 2, 9– 10] ^. Compared with the traditional cytotoxic chemotherapeutic agents such as cisplatin, EGFR-TKI has fewer side effects ^[ 11] ^. EGFR is highly expressed in the skin, therefore, skin toxicity is the most concerning issue for the EGFR-TKI treatment ^[ 6] ^. Several studies showed that the skin rash might be associated with the efficacy on EGFR-TKI ^[ 12] ^. Therefore, preventive or reactive treatment of skin rash caused by EGFR-TKI which improves the treatment compliance is essential to the patients with pancreatic cancer under EGFR-TKI treatment. Generally, topical steroid cream or ointment is prescribed for the treatment of EGFR inhibitor-associated skin rash, especially for acneiform rash. If patients experienced more severe skin rash, systemic dexamethasone or prednisolone is also prescribed ^[ 6, 13] ^. As the anti-tumor therapy undergoes a long-term process, the preventive or reactive treatment of skin rash also needs long-term persistence. However, tetracycline, doxycycline, minocycline and corticosteroid have obvious side effects such as the imbalance of intestinal flora, central obesity, osteoporosis, and skin thinning after long-time usage.

PZH has been used in China and Southeast Asia for centuries as a remedy for various types of diseases ^[ 14] ^. Studies have shown that PZH can alter the expression of IL-6 and STAT3, which indicates the therapeutic potential of PZH Unguentum Compositum in the treatment of inflammatory diseases of skin ^[ 14] ^. This is probably one of the effective mechanisms of PZH Unguentum Compositum for treating skin rash caused by EGFR-TKI. In this case report, our patient soon suffered skin rash after taking erlotinib which is consistent with previous reports, then PZH Unguentum Compositum was administered for external use. Surprisingly, skin rash caused by erlotinib was mitigated and eventually disappeared without any other side effects. The present case report suggests that PZH Unguentum Compositum may be a new option in treating the skin rash caused by EGFR-TKI such as erlotinib.

### Conclusions

While EGFR-TKIs have been proved to be an ideal class of antitumor drugs, skin rash remains its most concerning side effect. It is bothersome and sometimes affects the quality of life and treatment compliance. The drugs typically used in the EGFR inhibitor-associated skin rash, such as etracycline, doxycycline, minocycline and corticosteroid, all have obvious side effects after long-time use. TCM is considered to have fewer side effects, which is suitable for long-term external use. In this case, our experience highlights the successful treatment erlotinib-induced skin rash with PZH Unguentum Compositum without any observed side effects to date, improving the quality of life and treatment compliance.

In conclusion, PZH Unguentum Compositum provides an additional option for the patients suffering from skin toxicity caused by EGFR-TKIs.

###  

The authors thank the patient for his participation and his agreement to the publication of the report.
